# Supplementary material for: Nucleoporin 153 links nuclear pore complex to chromatin architecture by mediating CTCF and cohesin binding
Source: Nat Commun. 2020 May 25;11:2606. doi: 10.1038/s41467-020-16394-3 (PMC7248104; doi:10.1038/s41467-020-16394-3)
Supplement: Supplementary file 1 — Supplementary Information [file 41467_2020_16394_MOESM1_ESM.pdf]

Supplementary Information

Nucleoporin 153 links nuclear pore complex to chromatin architecture by mediating CTCF and cohesin binding

Kadota *et al.*

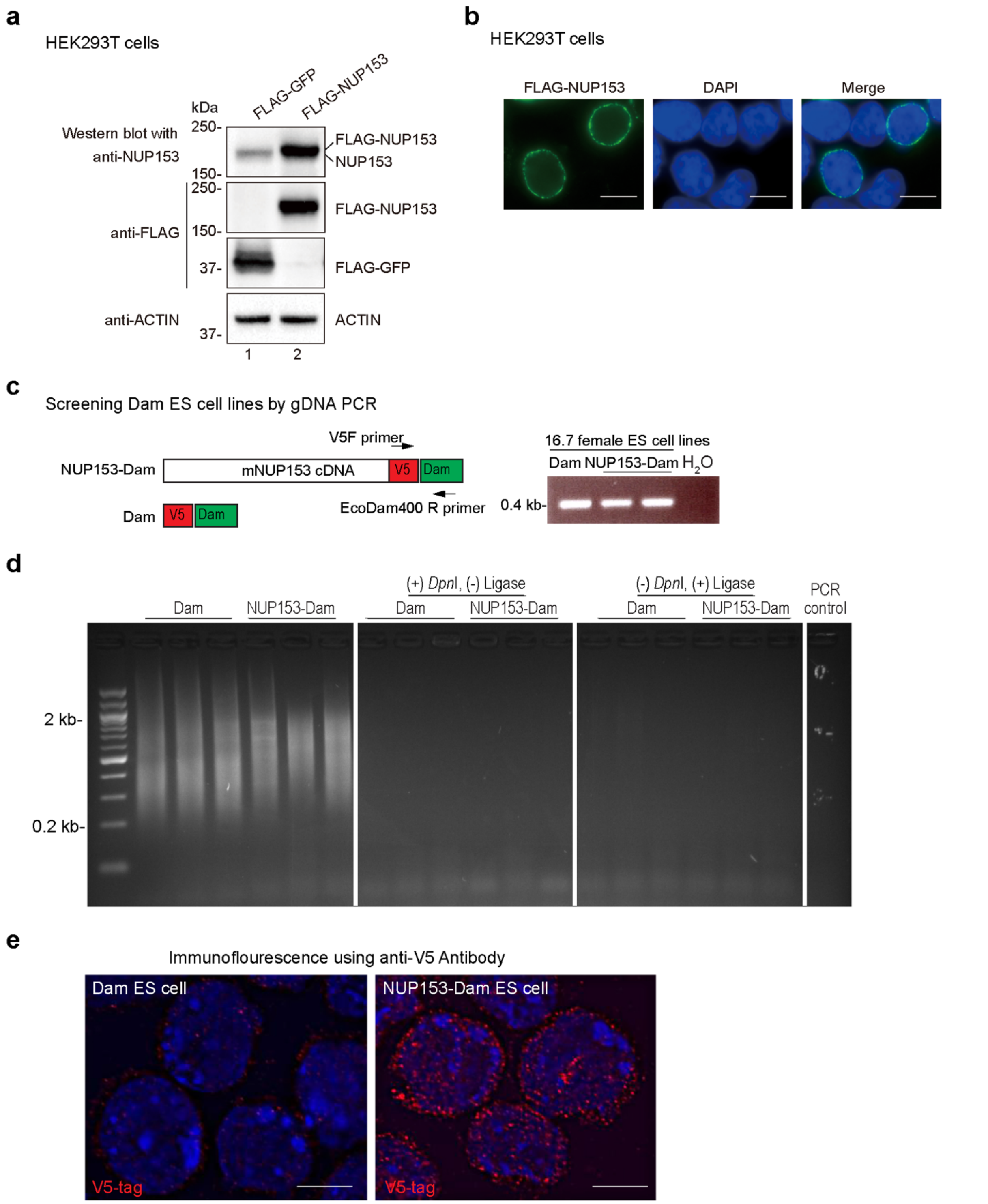

Supplementary Figure 1. FLAG-NUP153 expression in HEK293T cells and generation of NUP153-Dam mouse ES cell lines. (**a**) Western blot showing validation of FLAG-NUP153 expression. Whole cell extracts were prepared from FLAG-GFP- or FLAG-NUP153-expressing

HEK293T cells and were subjected to western blot using anti-NUP153, and anti-FLAG antibodies as indicated. ACTIN was used as an internal control. Source data are provided as a “Source Data file”. (**b**) Cellular localization of FLAG-NUP153 protein in HEK293T cells was determined by immunostaining. Scale bar, 10μm. (**c**) Mouse NUP153-cDNA (4.5 kb) (ATCC) was cloned into *Kpn* I and *Xho* I sites in pIND-(V5)-EcoDam plasmid [1] to generate NUP153-Dam plasmid. Female mouse ES cell line 16.7 were electroporated using NUP153-Dam or Dam only (pIND-(V5)-EcoDam) plasmids. ES clones were screened by genomic DNA (gDNA) PCR using a primer pair that amplifies a 0.4 kb fragment across V5-tag and Dam sequences. (**d**) Several ES cell clones were screened for Dam activity by gDNA PCR as previously described [1]. (**e**) Expression of NUP153-Dam fusion protein was determined by performing immunofluorescence using anti-V5 antibody in Dam only and NUP153-Dam mouse ES cells. Scale bar, 10μm.

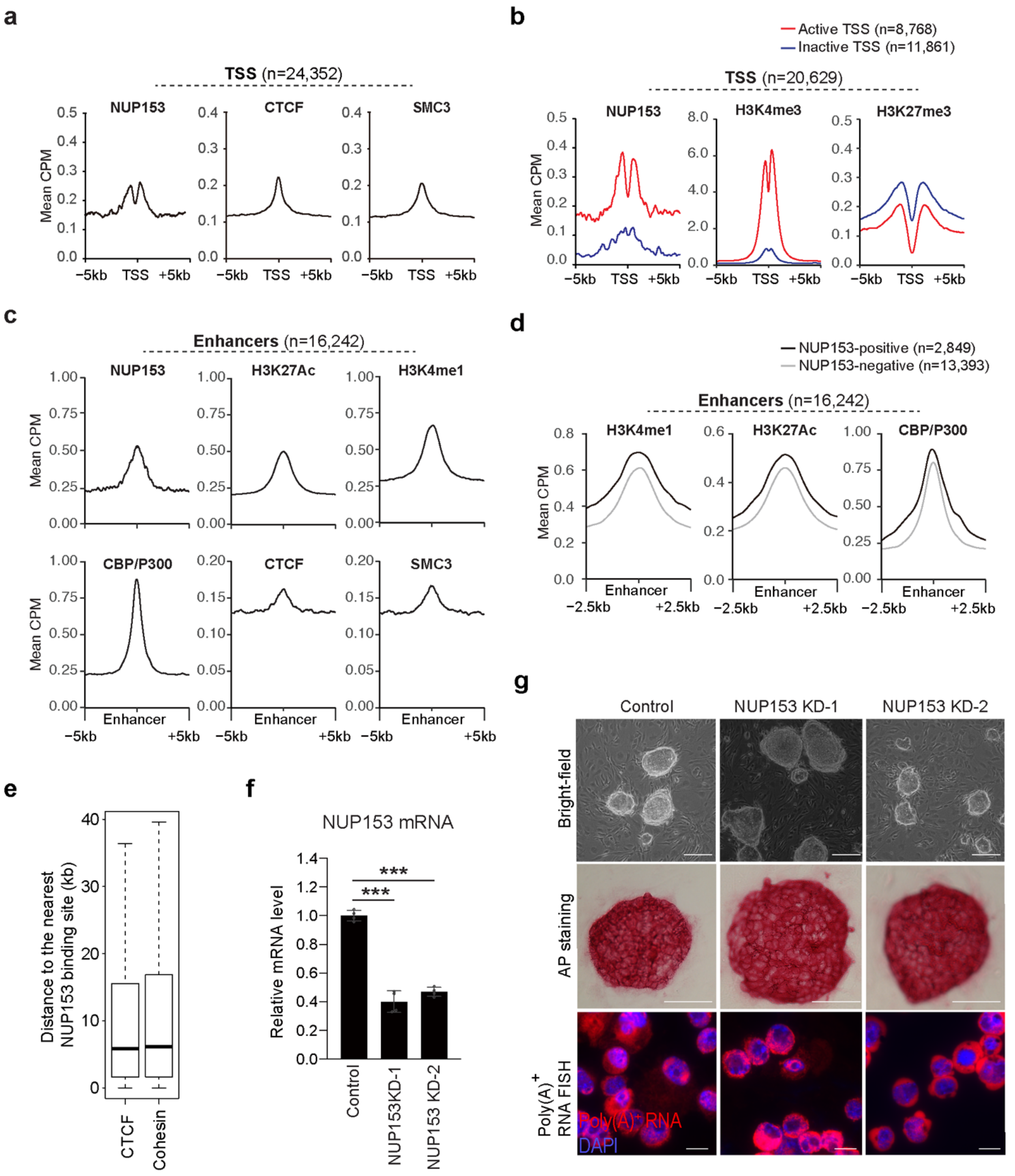

Supplementary Figure 2. Distribution of NUP153, CTCF, SMC3 and histone modifications across genetic elements in control (WT) mouse ES cells, and characteristics of NUP153 deficient mouse ES cells. (**a**) Metagene profiles showing mean CTCF, SMC3 and NUP153 binding at TSS (+/-5kb) (n=24,352). (**b**) Metagene profiles showing mean CTCF, SMC3 and NUP153 binding +/- 5kb of transcriptionally active (n=8,768) and inactive (n=11,861) TSS. (**c**)

Metagene profiles showing mean NUP153, H3K27Ac [2], H3K4me1 [3], CBP/P300 [4], CTCF, and SMC3 binding at enhancers (n=16,242)(+/-5kb). (**d**) Metagene profiles showing distribution of H3K27Ac [2], H3K4me1 [3], CBP/P300 [4] at NUP153-positive (n=2,849) and NUP153-negative (n=13,393) enhancers (+/-2.5kb) in control ES cells. (**e**) Distribution of CTCF or cohesin binding sites was evaluated to determine the median of their distance to the nearest NUP153 binding sites in mouse ES cells. CTCF and cohesin binding sites exhibit a median of ~5 kb distance to the nearest NUP153 binding sites. Boxed areas span the first to third quartiles, the center line represents the mean, and whiskers represent maximum or minimum observations. Outlier is not shown. (**f**) Real time RT-PCR showing relative NUP153 mRNA levels in control and NUP153 shRNA (KD-1, KD-2) lentivirus transduced ES cells. GAPDH mRNA level was used to normalize mRNA levels. Control vs KD-1, ***p=0.0000; control vs KD-2, ***p=0.0000. Mean mRNA levels ± standard error mean; two-tailed Student's t-test, n ≥ 3 independent experiments. Source data are provided as a "Source Data file". (**g**) Top panel, bright-field microscopy images showing typical pluripotent ES cell morphology of NUP153 deficient and control ES cells. Scale bar, 200μm; middle panel, immunostaining for alkaline phosphatase (AP) activity validates pluripotency of control and NUP153 KD ES cells. Scale bar, 100μm; bottom panel, oligo(dT)50-Cy3 RNA FISH for Poly(A)$^+$ RNA was performed in control and NUP153 KD ES cells to assess Poly(A)$^+$ RNA export. No significant defect in Poly(A)$^+$ RNA export was detected in NUP153 deficient cells. Scale bar, 20μm; n ≥ 3 independent experiments.

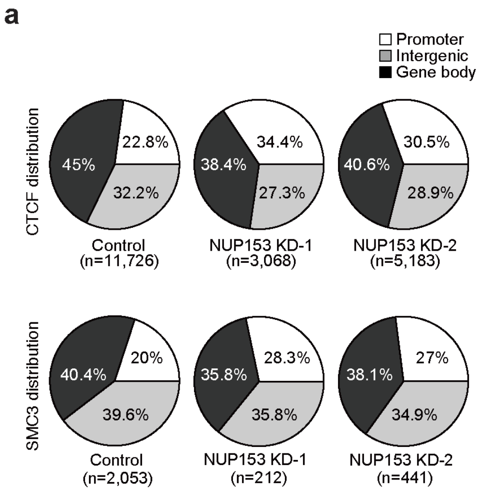

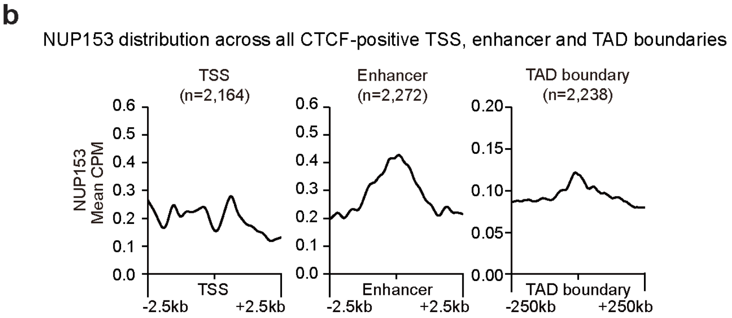

c

	Mean CTCF binding (CPM)					
	Control			NUP153 KD-1		
Group	TSS	Enhancer	TAD boundary	TSS	Enhancer	TAD boundary
I	0.86	0.37	0.18	0.52	0.26	0.14
II	0.63	0.19	0.17	0.52	0.19	0.13

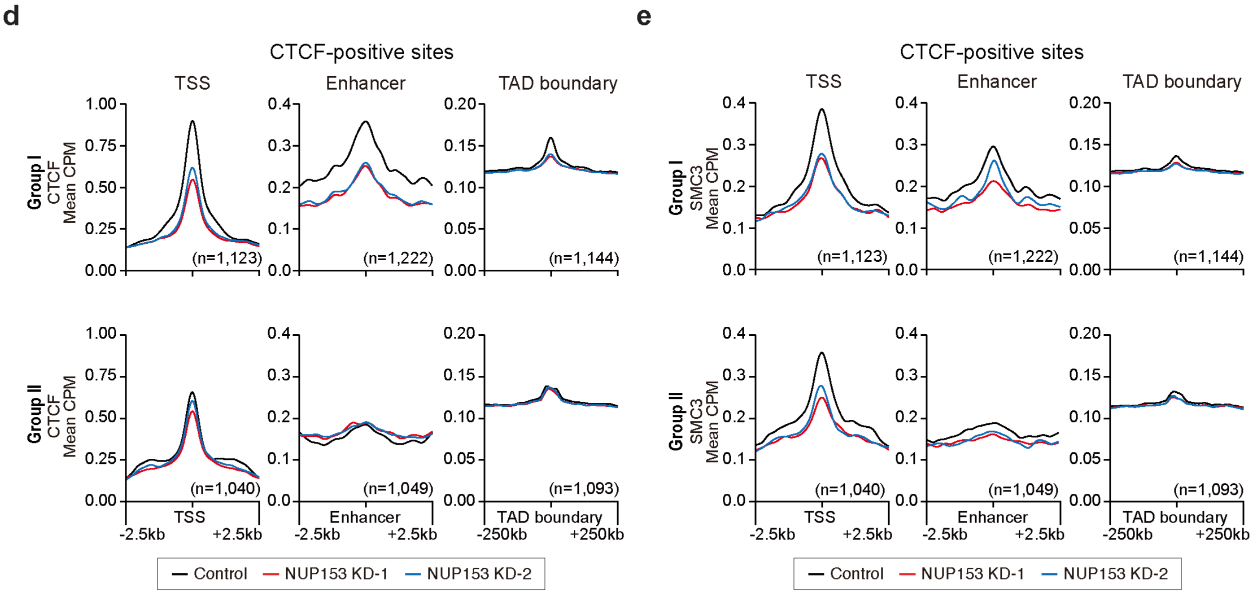

Supplementary Figure 3. Distribution of CTCF, SMC3 and NUP153 across genetic elements and CTCF-positive sites in mouse ES cells. (**a**) Distribution of CTCF and SMC3 sites across the indicated genetic elements in control and NUP153 KD-1 and KD-2 mouse ES cells. (**b**) Metagene profiles showing mean NUP153 binding at CTCF-positive TSS, enhancer and TAD boundaries. (**c**) Table showing mean CTCF binding at CTCF-positive TSS, enhancer and TAD boundaries in control and NUP153 KD ES cells. (**d-e**) Metagene profiles showing mean CTCF and SMC3 binding in control versus NUP153 KD cells at CTCF-positive Group I and Group II TSS, enhancer and TAD boundaries. Number of CTCF-positive sites for each Group is as indicated.

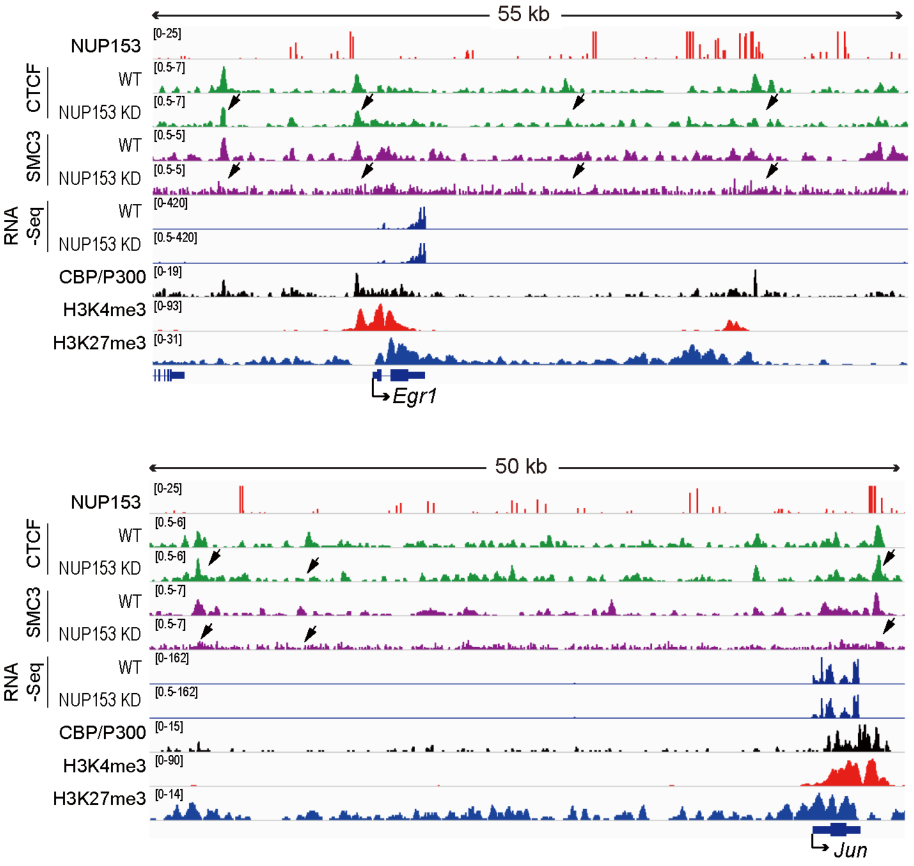

Supplementary Figure 4. IEG loci are NUP153 targets in mouse ES cells. CBP/P300, H3K4me3, H3K27me3, NUP153, CTCF, SMC3 ChIP-Seq, NUP153 DamID-Seq and RNA-Seq tracks are shown for IEG loci, *Egr1* (top panel), and *Jun* (bottom panel) in control and NUP153 KD ES cells. CBP/P300 [4], H3K4me3 and H3K27me3 [5] ChIP-Seq data were previously published.

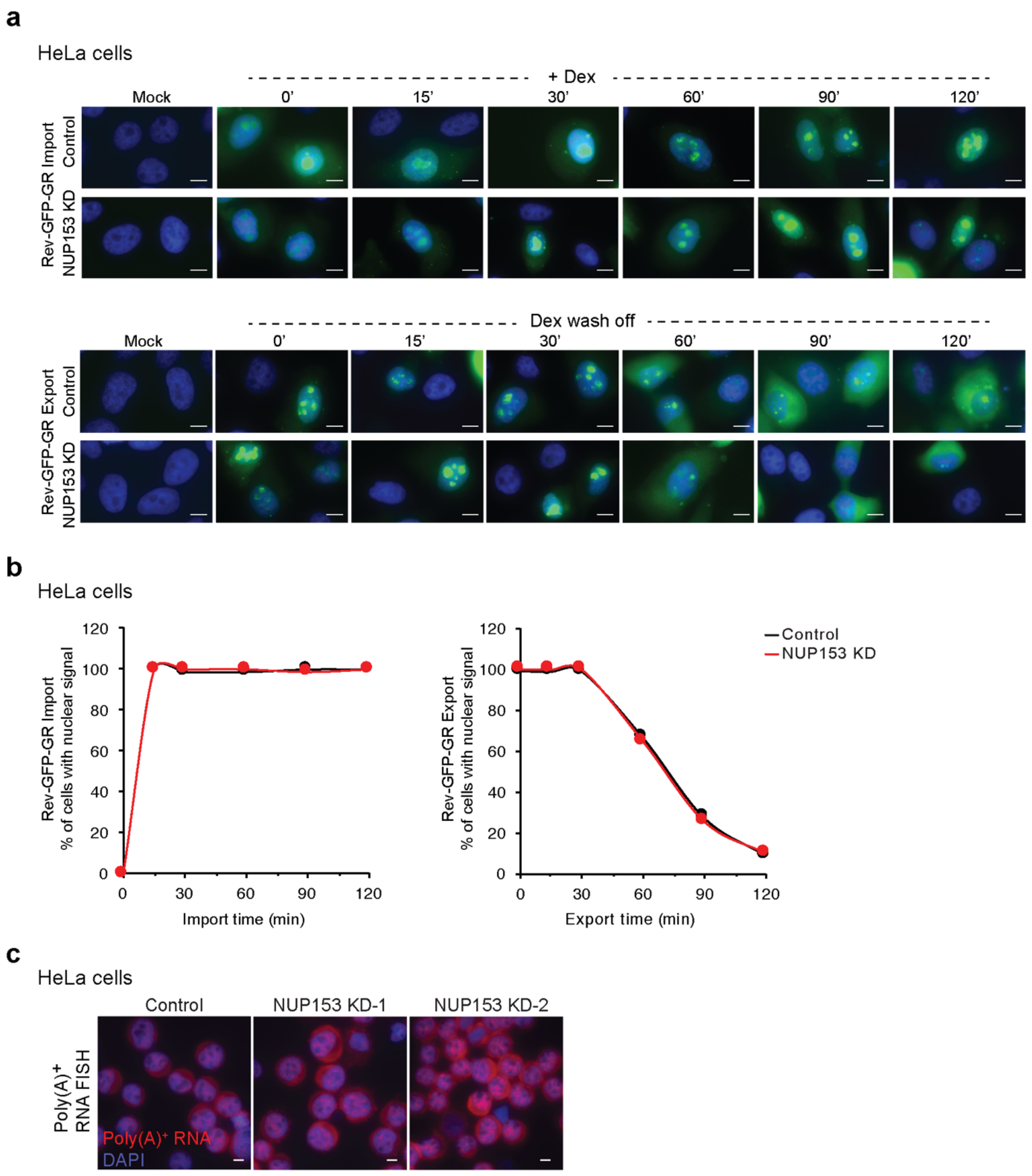

Supplementary Figure 5. Analyses of NUP153 deficient HeLa cells for nucleocytoplasmic trafficking. (**a**) Nuclear import and export were tracked using the dexamethasone responsive REV-GFP-GR construct (see Methods for more detailed information). Shown are representative images of control and NUP153 KD HeLa cells after dexamethasone (Dex) treatment or wash off at the indicated time points. To evaluate GR import, cells were treated with 250 mM Dex at the indicated times (n=68-84). To evaluate GR export, Dex was washed off after 120 mins

(considered zero (0) time point) (n=75). (**b**) Graphs showing nuclear import of REV-GFP-GR after Dex treatment (left) and export of REV-GFP-GR after Dex wash off (right). Values were calculated based on % of cells which show nuclear GR-GFP signal after Dex treatment, or Dex wash off at the indicated time points. Scale bar, 10μm. (**c**) Oligo(dT)50-Cy3 RNA FISH in control and NUP153 KD HeLa cells was performed to evaluate Poly(A)$^+$ RNA export. Scale bar, 10μm. Experiments were repeated twice. Source data are provided as a “Source Data file”.

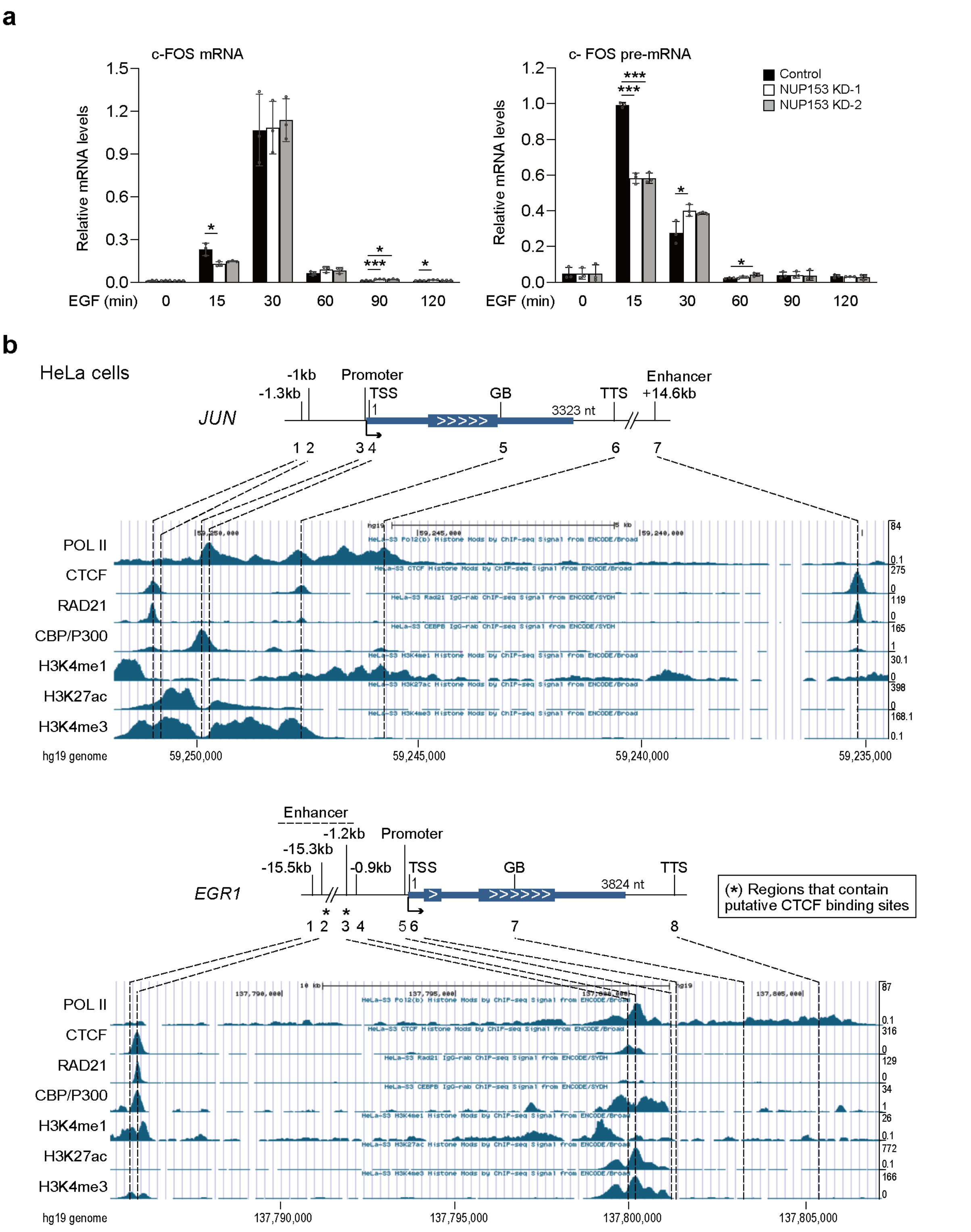

Supplementary Figure 6. Transcription and chromatin structure at the IEG loci in HeLa cells. (**a**) Real-time RT-PCR showing relative c-FOS mRNA and nascent mRNA levels in control and NUP153 KD HeLa cells in a time course dependent manner. *GAPDH* was used to normalize

mRNA levels. C-FOS mRNA levels at 15 min EGF (control vs KD-1, *p=0.0171), 90 min EGF (control vs KD-1, ***p=0.0009; control vs KD-2, *p=0.0145), 120 min EGF (control vs KD-1, *p=0.0139). C-FOS pre-mRNA levels at 15 min EGF (control vs KD-1, ***p=0.0000; control vs KD-2, ***p=0.0000), 30 min EGF (control vs KD-1, *p=0.0398), 60 min EGF (control vs KD-2, *p=0.0243). Values are mean ± standard deviation. Two-tailed Student's t-test, n ≥ 3 independent experiments. Source data are provided as a "Source Data file". (**b**) UCSC browser snapshots showing distribution of POL II, CTCF, cohesin subunit, RAD21, CBP/P300, and histone modifications, H3K4me1 and H3K27Ac, H3K4me3, across the *JUN* (top panel) and *EGR1* (bottom panel) loci in HeLa-S3 cells based on ENCODE ChIP-Seq datasets (see Methods for GEO information). ChIP-Seq read numbers are indicated at the right *y*-axis per data set. Human hg19 reference genome was used to analyze data sets. Asterisk (*) denotes sites that contain putative CTCF binding. Nt, nucleotide.

Distribution of *c-FOS* locus distance to nuclear periphery in HeLa cells

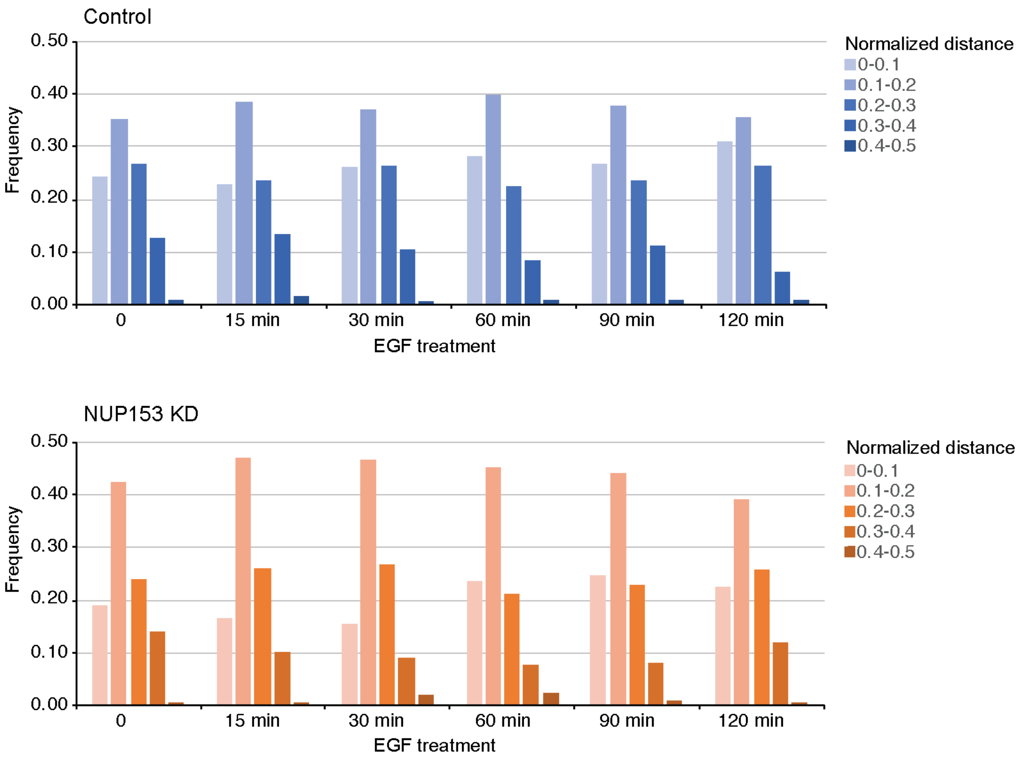

Supplementary Figure 7. Subnuclear position of *c-FOS* locus with respect to nuclear periphery in HeLa cells. Distribution of *c-FOS* locus distance to nuclear periphery in control and NUP153 KD HeLa cells was measured based on DNA FISH at the indicated time points +/- EGF (50 ng/ml). Frequencies at a normalized distance (ND) of 0.0-0.5 are shown. ND= *c-FOS* locus to periphery distance/cell diameter (d), where $d=(2x\text{nuclear area}/\pi)^{0.5}$. Control HeLa cells (Minus EGF, n=182; 15 min EGF, n=186; 30 min EGF, n=150; 60 min EGF, n=146; 90 min EGF, n=181; 120 min EGF, n=139); NUP153 KD HeLa cells (Minus EGF, n=66; 15 min EGF, n=138; 30 min EGF, n=170; 60 min EGF, n=106; 90 min EGF, n=237; 120 min EGF, n=170).

Supplementary References

1. Vogel MJ, Peric-Hupkes D, van Steensel B. Detection of in vivo protein-DNA interactions using DamID in mammalian cells. *Nat Protoc* **2**, 1467-1478 (2007).

2. Banaszynski LA*, et al.* Hira-dependent histone H3.3 deposition facilitates PRC2 recruitment at developmental loci in ES cells. *Cell* **155**, 107-120 (2013).

3. Ma Z, Swigut T, Valouev A, Rada-Iglesias A, Wysocka J. Sequence-specific regulator Prdm14 safeguards mouse ESCs from entering extraembryonic endoderm fates. *Nat Struct Mol Biol* **18**, 120-127 (2011).

4. Shen Y*, et al.* A map of the cis-regulatory sequences in the mouse genome. *Nature* **488**, 116-120 (2012).

5. Pinter SF*, et al.* Spreading of X chromosome inactivation via a hierarchy of defined Polycomb stations. *Genome Res* **22**, 1864-1876 (2012).
