# Supplementary material for: Nucleoporin 153 links nuclear pore complex to chromatin architecture by mediating CTCF and cohesin binding
Source: Nat Commun. 2020 May 25;11:2606. doi: 10.1038/s41467-020-16394-3 (PMC7248104; doi:10.1038/s41467-020-16394-3)
Supplement: Supplementary file 3 — Description of Additional Supplementary Information [file 41467_2020_16394_MOESM3_ESM.pdf]

Description of Additional Supplementary Files

File Name: Supplementary Data 1

Description: List of NUP153 peaks mapped by NUP153 DamID-Seq. To define high confidence binding sites (n=73,018), we calculated NUP153 enrichment in NUP153-Dam ES cell line over Dam-only ES cell line and selected sites that show FDR<0.05 by MACS2, Related to Figure 2, and Supplementary Figure 1 and 2.

File Name: Supplementary Data 2

Description: List of NUP153-positive A) TSS, B) enhancer, and C) TAD boundaries, Related to Figure 2.

File Name: Supplementary Data 3

Description: Number of global and genetic element-specific NUP153, CTCF and SMC3 binding sites in ES cell genome, Related to Figure 2.

File Name: Supplementary Data 4

Description: Mean CTCF or SMC3 binding (CPM) at CTCF-positive TSS, enhancer or TAD boundaries in control ES cells in comparison to NUP153 KD ES cells. Related to Figure 2, 3, and Supplementary Figure 3. *P*-values are determined by the two-sided Kolmogorov-Smirnov test.

File Name: Supplementary Data 5

Description: NUP153 binding, transcription, and bivalent state of genes that associate with CTCF-positive TSS, Related to Figure 2, 3, and Supplementary Figure 3.

File Name: Supplementary Data 6

Description: List of differentially regulated genes in NUP153 KD ES cells in comparison to control ES cell line. Normalization factors to scale the raw library sizes were calculated by upper-quartile normalization method. The two-sided likelihood ratio tests were performed by edgeR package in linear model. The fixed dispersion was calculated by spike-in with removed unwanted variation by RUVseq package. The

adjusted *p*-values (FDR) were calculated by edgeR with the default method. Significance was calculated based on fold change > 1.5 and FDR < 0.05, Related to Figure 3.

File Name: Supplementary Data 7

Description: Gene ontology (GO) analyses of differentially regulated genes in NUP153 KD mouse ES cells in comparison to control ES cells. GO terms that associated with the biological function of A) up regulated, B) down regulated and C) CTCF-positive Group I TSS associated genes are listed. *P*-values for hypergeometric distribution were calculated by clusterProfiler package. The adjusted *p*-values (FDR) were calculated by Benjamini & Hochberg method. Related to Figure 3 and Supplementary Figure 3.

File Name: Supplementary Data 8

Description: List of differentially expressed genes that associate with CTCF-positive TSS. Normalization factors to scale the raw library sizes were calculated by upper-quartile normalization method. Two-sided likelihood ratio tests were performed by edgeR package in linear model. The fixed dispersion was calculated by spike-in with removed unwanted variation by RUVseq package. The adjusted *p*-values (FDR) were calculated by edgeR with the default method. Significance was calculated based on fold change >0.5 and p-value<0.05, Related to Figure 2, 3, and Supplementary Figure 3.

File Name: Supplementary Data 9

Description: List of differentially regulated bivalent genes in NUP153 KD ES cells in comparison to control ES cell line. Normalization factors to scale the raw library sizes were calculated by upper-quartile normalization method. Two-sided likelihood ratio tests were performed by edgeR package in linear model. The fixed dispersion was calculated by spike-in with removed unwanted variation by RUVseq package. The adjusted p-values (FDR) were calculated by edgeR with the default method. Significance was calculated based on fold change >1.5 and FDR < 0.05, Related to Figure 3.

File Name: Supplementary Data 10

Description: Primer sets used for RT and ChIP real-time PCR, Related to Figure 4, 5, 6, and Supplementary Figure 6.
