# Supplementary material for: Nucleoporin 153 links nuclear pore complex to chromatin architecture by mediating CTCF and cohesin binding
Source: Nat Commun. 2020 May 25;11:2606. doi: 10.1038/s41467-020-16394-3 (PMC7248104; doi:10.1038/s41467-020-16394-3)
Supplement: Supplementary file 14 — Reporting summary [file 41467_2020_16394_MOESM14_ESM.pdf]

nature research

Corresponding author(s): Eda Yildirim

Last updated by author(s): 04-19-2020

Reporting Summary

Nature Research wishes to improve the reproducibility of the work that we publish. This form provides structure for consistency and transparency in reporting. For further information on Nature Research policies, see Authors & Referees and the Editorial Policy Checklist .

Statistics

For all statistical analyses, confirm that the following items are present in the figure legend, table legend, main text, or Methods section.

n/a	Confirmed	
☐	☒	The exact sample size (n) for each experimental group/condition, given as a discrete number and unit of measurement
☐	☒	A statement on whether measurements were taken from distinct samples or whether the same sample was measured repeatedly
☐	☒	The statistical test(s) used AND whether they are one- or two-sided<br>*Only common tests should be described solely by name; describe more complex techniques in the Methods section.*
☒	☐	A description of all covariates tested
☒	☐	A description of any assumptions or corrections, such as tests of normality and adjustment for multiple comparisons
☒	☐	A full description of the statistical parameters including central tendency (e.g. means) or other basic estimates (e.g. regression coefficient) AND variation (e.g. standard deviation) or associated estimates of uncertainty (e.g. confidence intervals)
☐	☒	For null hypothesis testing, the test statistic (e.g. F, t, r) with confidence intervals, effect sizes, degrees of freedom and P value noted<br>*Give P values as exact values whenever suitable.*
☒	☐	For Bayesian analysis, information on the choice of priors and Markov chain Monte Carlo settings
☒	☐	For hierarchical and complex designs, identification of the appropriate level for tests and full reporting of outcomes
☐	☒	Estimates of effect sizes (e.g. Cohen's d, Pearson's r), indicating how they were calculated

Our web collection on statistics for biologists contains articles on many of the points above.

Software and code

Policy information about availability of computer code

Data collection	Flourescent in situ hybridization and immunoflourescence data were acquired with a Leica DM5500B microscope and Leica DFC365 FX CCD camera. Image analysis was performed using ImageJ (v2.0.0) software.
Data analysis	Software used has been described in "Methods". Please also find a list below:<br>Bcl2Fastq (v2)<br>Bowtie2 (v.2.2.5)<br>ChIPpeakAnno (v.3.19.5)<br>Cufflinks (v 2.1.1)<br>EdgeR (v.3.24.0)<br>FeatureCounts (v.1.6.1)<br>ImageJ (v.2.0.0)<br>MACS2 (v.2.1.0)<br>PeptideProphet algorithm<br>Picard (v.1.91)<br>RUVseq (v.1.16.0)<br>Samtools (v.1.5)<br>Scaffold (v4.4)<br>TopHAT (v.2.1.1)<br>TrackViewer (v. 1.23.2)<br>Trim Galore (v.0.4.1)

For manuscripts utilizing custom algorithms or software that are central to the research but not yet described in published literature, software must be made available to editors/reviewers. We strongly encourage code deposition in a community repository (e.g. GitHub). See the Nature Research guidelines for submitting code & software for further information.

Data

Policy information about availability of data

All manuscripts must include a data availability statement. This statement should provide the following information, where applicable:

- Accession codes, unique identifiers, or web links for publicly available datasets
- A list of figures that have associated raw data
- A description of any restrictions on data availability

Gene expression profiles, DamID-Seq and ChIP-Seq datasets have been deposited at GEO [https://www.ncbi.nlm.nih.gov/geo/query/acc.cgi?acc=GSE135647], 'GSE135647'. Proteomics data have been deposited to the ProteomeXchange Consortium via the PRIDE partner repository [https://www.ebi.ac.uk/pride/] with the Project ID: PXD015441. The source data underlying Figures. 1a-b, 1d, 4a-d, 5, 6a-d, 7a-b and Supplementary Figures. 1a, 2f, 5a-b, 6a, 7 are provided as a Source Data file. All other relevant data supporting the key findings of this study are available within the article and its Supplementary Information files from the corresponding author upon reasonable request. ENCODE HeLa-S3 ChIP-Seq data sets for POL II (GEO: GSM733759), CTCF (GEO: GSM733785), RAD21 (GEO: GSM935571), CBP/P300 (GEO: GSM935553), H3K4me1 (GEO: GSM798322), H3K27Ac (GEO: GSM733684), and H3K4me3 (GEO: GSM733682) were utilized to examine chromatin structure across the JUN and EGR1 genes (Supplementary Figure 6B) using Human hg19 as a reference genome. Promoters are defined by gene start sites downloaded from UCSC Genome Browser goldenPath/mm10/database/knownGene. Chromatin structure at the transcriptionally active vs inactive TSS was validated using previously published H3K4me3 and H3K27me3 ChIP-Seq, respectively (GEO: GSE36905). Enhancers were defined by utilizing the previously published ChIP-Seq data sets and determining the overlapping region of peaks with at least two enhancer specific markers including CBP/P300 (GEO: GSE29184), H3K4me1 (GEO: GSE25409) or H3K27Ac (GEO: GSE42152). Mouse ES cell normalized 40kb HiC Matrices (mm9) were downloaded from http://chromosome.sdsc.edu/mouse/hi-c/download.html.

Field-specific reporting

Please select the one below that is the best fit for your research. If you are not sure, read the appropriate sections before making your selection.

☒ Life sciences ☐ Behavioural & social sciences ☐ Ecological, evolutionary & environmental sciences

For a reference copy of the document with all sections, see nature.com/documents/nr-reporting-summary-flat.pdf

Life sciences study design

All studies must disclose on these points even when the disclosure is negative.

Sample size	No sample size calculation was performed. Two different shRNAs were used to determine NUP153 knockdown phenotype and investigate role of NUP153 in chromatin architecture and transcription. RNA-Seq and ChIP-Seq (CTCF, SMC3) were performed on mouse embryonic stem (ES) cells that were transduced with scramble (control) and two different NUP153-specific shRNA (NUP153 knockdown) virus particles. NUP153, CTCF, SMC3, POL II ChIP in HeLa cells were performed using HeLa cells transfected with either scramble shRNA (control) or two different NUP153-specific shRNA plasmids (NUP153 KD). See 'Methods' for shRNA sequence information. Eco-Dam and NUP153 DamID Seq was performed in one biological replicate. In immuno-DNA-FISH experiments, number of cells counted was listed in each figure. Mass-spec was performed in FLAG-NUP153 expressing HEK-293 cells. In Alkaline Phosphatase staining and PolyA+ RNA FISH experiments, >100 cells were examined. Examining >100 cells increased the technical reproducibility of each experiment. Sample sizes for the other experiments were chosen on the basis of preliminary experiments and literature.
Data exclusions	No data exclusions.
Replication	RNA-Seq and ChIP-Seq (CTCF, SMC3) were performed in one control and two different NUP153 specific shRNA infected mouse ES cells. NUP153, CTCF, SMC3, and POL II ChIP real-time PCR analyses in HeLa cells were performed using HeLa cells transfected with either scramble shRNA (control) or two different NUP153-specific shRNA plasmids and were repeated more than three times. Real-time PCR analyses of transcription at specific loci were repeated more than three times. Eco-Dam and NUP153 DamID Seq was performed in one biological replicate and performed once. Co-IP experiments were repeated at least twice or three times. Immuno-DNA-FISH was performed with at least two biological replicates using control and NUP153 knockdown HeLa cells. Experiments were successfully reproduced as reported in the source data file.
Randomization	This work does not involve participant groups or animals so randomization was not needed for the study.
Blinding	This work does not involve participant groups or animals so blinding was not relevant for this kind of study

Reporting for specific materials, systems and methods

We require information from authors about some types of materials, experimental systems and methods used in many studies. Here, indicate whether each material, system or method listed is relevant to your study. If you are not sure if a list item applies to your research, read the appropriate section before selecting a response.

Materials & experimental systems

n/a	Involved in the study	
☐	☒	Antibodies
☐	☒	Eukaryotic cell lines
☒	☐	Palaeontology
☐	☒	Animals and other organisms
☒	☐	Human research participants
☒	☐	Clinical data

Methods

n/a	Involved in the study	
☐	☒	ChIP-seq
☒	☐	Flow cytometry
☒	☐	MRI-based neuroimaging

Antibodies

Antibodies used

Goat polyclonal anti-IgG(H+L)-Alexa 488 Thermo Fisher Scientific Cat#A-11008, RRID:AB_143165
Goat polyclonal anti-IgG(H+L)-Alexa 488 Thermo Fisher Scientific Cat#A32723, RRID:AB_2633275
Mouse monoclonal anti-alpha-Tubulin Santa Cruz Biotechnology Cat#sc-5286; RRID:AB_628411
Mouse monoclonal anti-Flag M2 Sigma-Aldrich Cat#F1804; RRID:AB_262044
Mouse monoclonal anti-Nup153 Abcam Cat#ab24700; RRID:AB_2154467
Mouse monoclonal anti-V5 Tag Thermo Fisher Scientific Cat# R960-25, RRID:AB_2556564
Rabbit monoclonal anti-Rpb1 NTD Cell Signaling Technology Cat#14958; RRID:AB_2687876
Rabbit polyclonal anti-CTCF Millipore Cat#07-729; RRID:AB_441965
Rabbit polyclonal anti-CTCF Cell Signaling Technology Cat#2899S; RRID:AB_2086794
Rabbit polyclonal anti-GAPDH Sigma-Aldrich Cat#G9545; RRID:AB_796208
Rabbit polyclonal anti-Histone H3 Abcam Cat#ab1791; RRID:AB_302613
Rabbit polyclonal anti-IgG(H+L)-Alexa 555 Thermo Fisher Scientific Cat#A-21427, RRID:AB_2535848
Rabbit polyclonal anti-Lamin B1 Abcam Cat# ab16048, RRID:AB_443298
Rabbit polyclonal anti-RAD21 Abcam Cat # ab992, RRID:AB_2176601
Rabbit polyclonal anti-SMC1A Bethyl Cat#A300-055A; RRID:AB_2192467
Rabbit polyclonal anti-SMC3 Abcam Cat#ab9263; RRID:AB_307122

Validation

All the antibodies were already validated and used according to the manufacturer instructions. See ID of antibodies. Information on the methods of validation and applications for each antibody are available from http://antibodyregistry.org/
We listed the antibody registry links to the antibodies used in this study below:
AB_143165; https://antibodyregistry.org/search.php?q=AB_143165
AB_2633275; https://antibodyregistry.org/search?q=AB_2633275
AB_628411; https://antibodyregistry.org/search?q=AB_628411
AB_262044; https://antibodyregistry.org/search?q=AB_262044
AB_2154467; https://antibodyregistry.org/search?q=AB_2154467
AB_2556564; https://antibodyregistry.org/search?q=AB_2556564
AB_2687876; https://antibodyregistry.org/search?q=AB_2687876
AB_441965; https://antibodyregistry.org/search?q=AB_441965
AB_2086794; https://antibodyregistry.org/search?q=AB_2086794
AB_796208; https://antibodyregistry.org/search?q=AB_796208
AB_302613; https://antibodyregistry.org/search?q=AB_302613
AB_2535848; https://antibodyregistry.org/search?q=AB_2535848
AB_443298; https://antibodyregistry.org/search?q=AB_443298
AB_2176601; https://antibodyregistry.org/search?q=AB992
AB_2192467; https://antibodyregistry.org/search.php?q=AB_2192467
AB_307122; https://antibodyregistry.org/search?q=AB_307122

Eukaryotic cell lines

Policy information about cell lines

Cell line source(s)

Female wild-type mouse embryonic stem cell line EL16.7 is a gift from Dr. Jeannie T. Lee (Harvard) and the cell line have been described, as referenced in the manuscript. HeLa and HEK293T cells were obtained from the American Tissue Collection Center (ATCC, Manassas, VA, USA) through the Duke University Cancer Center Facilities. Mouse ES cells were cultured on gamma-irradiated mouse embryonic fibroblasts (MEFs) that were isolated in house from Tg(DR4)1Jae/J mice (The Jackson Laboratory).

Authentication

HEK293T and HeLa cell lines were authenticated by the Duke University DNA Analysis Facility using polymorphic short tandem repeats by utilizing GenePrint 10 kit (Promega). Next generation sequencing supports genotype of mouse ES cells.

Mycoplasma contamination

All original cell stocks had been tested and were found negative.

Commonly misidentified lines
(See ICLAC register)

No commonly misidentified cell lines were used.

Animals and other organisms

Policy information about studies involving animals; ARRIVE guidelines recommended for reporting animal research

Laboratory animals	*For laboratory animals, report species, strain, sex and age OR state that the study did not involve laboratory animals.*
Wild animals	*Provide details on animals observed in or captured in the field; report species, sex and age where possible. Describe how animals were caught and transported and what happened to captive animals after the study (if killed, explain why and describe method; if released, say where and when) OR state that the study did not involve wild animals.*
Field-collected samples	*For laboratory work with field-collected samples, describe all relevant parameters such as housing, maintenance, temperature, photoperiod and end-of-experiment protocol OR state that the study did not involve samples collected from the field.*
Ethics oversight	*Identify the organization(s) that approved or provided guidance on the study protocol, OR state that no ethical approval or guidance was required and explain why not.*

Note that full information on the approval of the study protocol must also be provided in the manuscript.

ChIP-seq

Data deposition

- [x] Confirm that both raw and final processed data have been deposited in a public database such as GEO.
- [x] Confirm that you have deposited or provided access to graph files (e.g. BED files) for the called peaks.

Data access links
May remain private before publication.

ChIP-Seq, DamID-Seq data access link: https://www.ncbi.nlm.nih.gov/geo/query/acc.cgi?acc=GSE135647.

Files in database submission

5_100622_FC621HLAAXX_1_fastq.txt
5_100622_FC621HLAAXX_2_fastq.txt
7_100622_FC621HLAAXX_1_fastq.txt
7_100622_FC621HLAAXX_2_fastq.txt
Input_sh002_d0_16_7_ESC_L002_R1_001.fastq
Input_sh38_d0_16_7_ESC_L002_R1_001.fastq
Input_sh98_d0_16_7_ESC_L002_R1_001.fastq
Smc3_sh002_d0_16_7_ESC_S14_L004_R1_001.fastq
Smc3_sh38_d0_16_7_ESC_S15_L004_R1_001.fastq
Smc3_sh98_d0_16_7_ESC_S16_L004_R1_001.fastq
Ctcf_sh002_d0_16_7_ESC_S11_L003_R1_001.fastq
Ctcf_sh38_d0_16_7_ESC_S12_L003_R1_001.fastq
Ctcf_sh98_d0_16_7_ESC_S13_L003_R1_001.fastq

Genome browser session
(e.g. UCSC)

Not applicable.

Methodology

Replicates	ChIP-Seq (CTCF, SMC3) were performed in one control and two different NUP153 specific shRNA infected mouse ES cells. Eco-Dam and NUP153 DamID Seq was performed in one biological replicate and performed once.
Sequencing depth	Sequencing was done at 50 bp single-end and generated ~110x106 reads per library.
Antibodies	Rabbit monoclonal anti-Rpb1 NTD Cell Signaling Technology Cat#14958; RRID:AB_2687876<br>Rabbit polyclonal anti-CTCF Cell Signaling Technology Cat#2899S; RRID:AB_2086794<br>Rabbit polyclonal Rabbit polyclonal anti-SMC3 Abcam Cat#ab9263; RRID:AB_307122
Peak calling parameters	The ChIP-Seq peaks were called by MACS2 (v 2.1.0, with --pvalue 1e-5). The read coverages were quantified by the signal in reads per million per base pair https://github.com/BradnerLab/pipeline/blob/master/bamToGFF.py with parameters -m 500 -r -d. Metagene plots were used to display the average ChIP-seq signal across related regions of interest for enhancers and TSS separately. The average profile (metagene) was calculated by the mean of ChIP-seq signal profiles across the related regions of interest. For each metagene plot, the profile is displayed in rpm/bp in a ±2.5 or 5 kb region centered on the regions of interest. The number of enhancers or TSS were noted in the title of plots. DamID-Seq reads were mapped with bowtie2 (.2.5, with parameters --very-sensitive) to mouse genome (UCSC mm10). The mapped reads were filtered by MAPQ greater than 30 by samtools (v 1.5) and filtered by GATC in 5' ends. The peaks were called by MACS2 (v 2.1.0, with -q 0.05). To determine distribution of NUP153-DamID peaks across the genetic elements in mouse ES cells we used the following criterion. Promoters (-2kb from TSS to +100 bp from TSS); GB (+100bp from TSS to +1kb from TTS); Intergenic sites (< -2kb from TSS and >+1kb from TTS). TSS, transcription start site; GB, gene body; TTS, transcription termination site.

Data quality

The ChIP-Seq peaks were called by MACS2 (v 2.1.0, with --pvalue 1e-5). DamID-Seq reads were mapped with bowtie2 (2.2.5, with parameters --very-sensitive) to mouse genome (UCSC mm10). The mapped reads were filtered by MAPQ greater than 30 by samtools (v 1.5) and filtered by GATC in 5’ ends. The peaks were called by MACS2 (v 2.1.0, with -q 0.05).

Software

ChIP-Seq reads were trimmed by Trim Galore (0.4.1, with -q 15) and then mapped with bowtie2 (2.2.5, with parameters --very-sensitive) to mouse genome (UCSC mm10). The mapped reads were filtered by MAPQ greater than 30 by samtools (v 1.5) and duplicated reads were removed by picard (v 1.91). DamID-Seq data was filtered by GATC in 5’ ends. The peaks were called by MACS2 (v 2.1.0, with -q 0.05). The read coverages were quantified by the signal in reads per million per base pair deepTools:bamCoverage with parameters -m 500 -r -d.
